# Space‐Time‐Coding Metasurfaces Enabling Programmable Range‐Angle‐Dependent Energy Auto‐Scanning

**DOI:** 10.1002/nap2.70204

**Published:** 2026-07-16

**Authors:** Yuzhen Chen, Xiaoyi Wang, Guo‐Min Yang, Ya‐Qiu Jin

**Affiliations:** ^1^ Key Laboratory for Information Science of Electromagnetic Waves (MoE) Fudan University Shanghai China; ^2^ College of Electronic and Information Engineering Tongji University Shanghai China

## Abstract

Dynamic regulation of electromagnetic energy distribution in space plays a pivotal role in fields like radar detection and wireless communications. However, the state‐of‐the‐art solution based on antenna array requires transmit/receive (T/R) modules per radiating element, causing exponential cost scaling and high power consumption. While reconfigurable metasurfaces have been demonstrated to enable real‐time beam scanning in prior works, they can only adjust beam direction and lack the ability to tune energy distribution across different distances. This paper presents a theory for space‐time‐coding metasurface (STCM) enabled spatiotemporal energy auto‐scanning. By applying identical time‐varying modulation signals with time delays to each column of the STCM, the reflected energy distribution can be made angle‐range‐time‐dependent. Based on this principle, a novel positioning system is designed, enabling decoupled estimation of a target's angle and distance relative to the metasurface. For experimental validation, a 2‐bit metasurface prototype is fabricated and tested. Experimental results align with theoretical analysis, confirming STCM's functional efficacy and deployment potential for applications in radar detection, wireless communications, and related fields.

## Graphical abstract

This paper presents a novel energy auto‐scanning system based on a space‐time‐coding metasurface. By applying progressive time‐delayed, time‐varying phase modulation, the system generates non‐uniform, temporally evolving electromagnetic field distributions across both angle and range. Furthermore, a single‐channel positioning system leveraging the MUSIC algorithm is demonstrated to estimate target angles and ranges using the proposed platform.

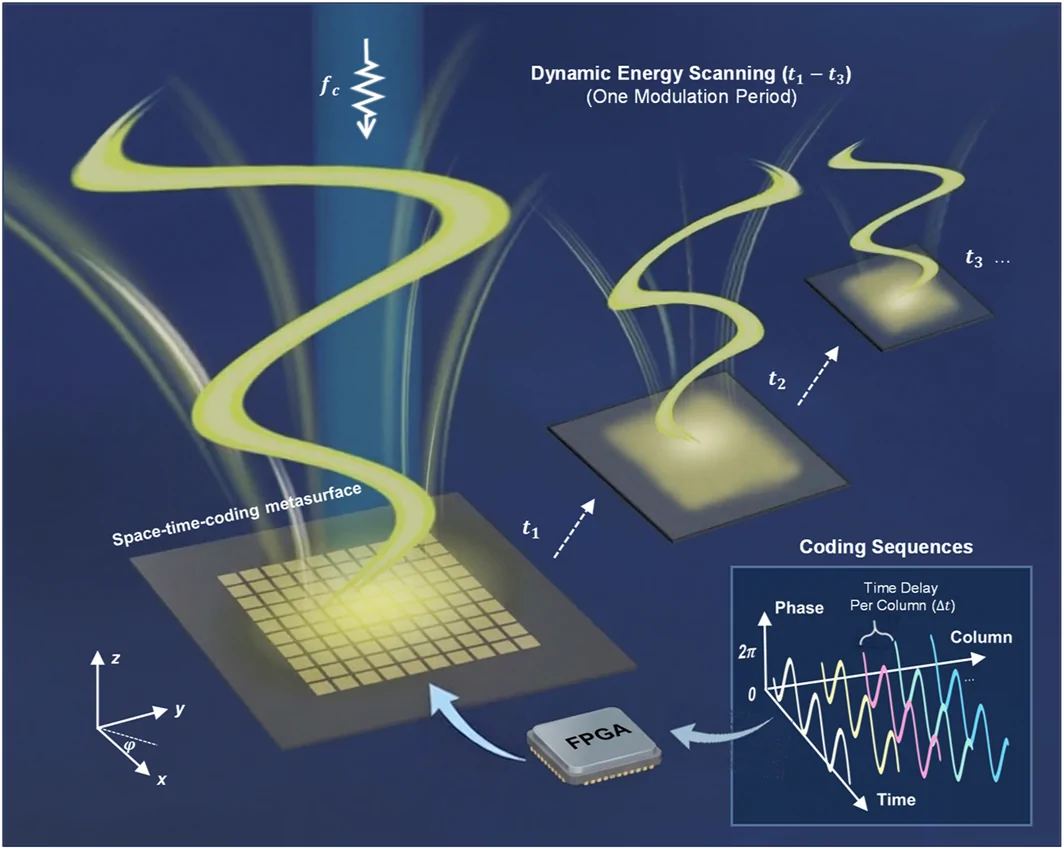

## 1 Introduction

Electromagnetic (EM) energy scanning technology serves as a pivotal innovation across scientific and industrial fields. By harnessing interactions between EM waves and materials, it enables non‐invasive probing [1], quantitative characterization, and high‐fidelity imaging of structures [2, 3]—capabilities foundational to applications spanning defect detection [4, 5, 6, 7], thickness monitoring [8, 9, 10, 11], and notably, real‐time spatial localization [12, 13, 14]. The proliferation of 5G/6G networks and dense IoT ecosystems further extends its utility, particularly in scenarios demanding precise electromagnetic field mapping for device coordination and positioning [15, 16, 17]. Advances in computational imaging and machine learning continue to refine this technology's resolution and efficiency, cementing its role in critical domains from structural health diagnostics [18, 19] to next‐generation spatial intelligence systems [20].

Current energy‐scanning technologies exhibit profound and multidimensional limitations. In its most fundamental manifestation, mechanical scanners and phased arrays achieve angular coverage through controlled aperture steering or phase gradient manipulation, but intrinsically lack range‐dimension control [21, 22, 23]. This inherent limitation fundamentally constrains their efficacy in suppressing range‐dependent interference and executing other range‐discriminative operations. Frequency diverse arrays (FDAs) realize two‐dimensional angle‐range joint scanning by feeding the array elements with a uniform frequency offset, which underpins their capability for joint space‐time beamforming [24, 25]. Nevertheless, the performance of FDAs is inherently limited by the unresolvable angle‐range coupling effect that arises specifically under the uniform frequency offset excitation scheme, which not only degrades the angle‐range resolution but also introduces severe localization ambiguities in target detection and parameter estimation [13, 26, 27]. Additionally, FDA systems suffer from significant hardware complexity, as they require providing distinct frequency excitations to each element of the antenna array. As an alternative, multiple‐input multiple‐output (MIMO) systems have been developed, where orthogonal waveforms transmitted by individual antennas or subarrays enable decoupled angle and range estimation in theory via ideal cross‐correlation properties [28, 29, 30, 31]. However, practical implementation faces fundamental limitations stemming from two primary challenges: the inherent design complexity of achieving mutual waveform orthogonality under shared time‐frequency resources, while hardware‐induced distortions progressively degrade orthogonality and compromise cross‐correlation integrity. To circumvent this constraint, space‐time coding array (STCA) is proposed, achieving range‐angle dependent energy auto‐scanning by introducing progressive time delays between adjacent elements [32, 33, 34, 35]. This architecture provides full spatial coverage using a single waveform, thereby eliminating requirements for sophisticated advanced frequency synthesis circuits or waveform generation systems.

The aforementioned solutions, which employ array‐based methodologies, inherently depend on sophisticated multi‐channel feeding networks that impose prohibitive constraints, including high power consumption, physical bulk, and reduced operational flexibility. Consequently, a critical unmet need persists: the development of a compact, low‐cost platform capable of autonomously scanning the total electromagnetic energy landscape while providing decoupled control over both angular and range coordinates. Recently, metasurfaces—artificially engineered thin‐layer structures with customizable electromagnetic properties—have revolutionized precise wave manipulation beyond conventional antenna arrays, enabling transformative advances across fundamental physics and wireless systems [36, 37, 38, 39, 40]. However, conventional metasurfaces inherently depend on fixed modulation signals, confining their functionality only to quasi‐static operation in practical scenarios due to millisecond‐scale transition latencies. To overcome this limitation, space‐time‐coding metasurfaces (STCMs) have emerged as a dynamic wave control platform [41, 42, 43, 44, 45]. STCMs employ predefined temporal coding sequences to dynamically reconfigure wavefronts. Unlike traditional antenna arrays, STCMs provide advantages such as simplified structures, versatile modulation, low cost, high efficiency, and diverse functionalities. Building upon this foundation, researchers have explored innovative STCM‐based applications such as sensing and anti‐sensing [46, 47, 48], and energy auto‐scanning. For instance, the authors in ref. 49 proposed an asynchronous time‐coded metasurface inspired by the FDA concept, wherein the coding sequences have different frequencies for individual metasurface elements. While this system enables joint energy auto‐scanning across range and angular domains, a critical implementation challenge arises from the technical complexity and hardware cost of applying different modulation frequencies to individual metasurface elements. More importantly, this system inherits the fundamental limitation of conventional FDAs—the coupling between range and angle measurements—which limits its applications in accurate radar positioning and detection.

To overcome these limitations, this work introduces a novel synchronous STCM‐based range‐angle‐dependent energy auto‐scanning, which shares same coding frequency in each metasurface element and enables simultaneous and decoupled estimation of target angle and range information. This functionality is realized by applying identical periodic nonlinear modulation signals with progressive time delays across adjacent metasurface columns. This configuration generates dynamic wavefronts, facilitating flexible spatial redistribution of the total reflected electromagnetic energy. Furthermore, a novel method for spatiotemporal electromagnetic energy trajectory design is proposed and verified by theoretical derivation and simulation. A fabricated 10 × 10 2‐bit STCM prototype operating at 5 GHz is employed to experimentally validate the principle of energy scanning in both angular and range dimensions. In addition, a single‐channel positioning system leveraging this mechanism achieves simultaneous target angle and distance detection is designed and measured. The proposed auto‐scanning metasurfaces extend the functionality of STCMs from basic wave modulation to actively directing the spatiotemporal distribution of energy, thereby enabling a more precise paradigm for intelligent electromagnetic control.

## 2 Results

### 2.1 Working Principle of Range‐Angle‐Dependent Energy Auto‐Scanning

Figure 1 illustrates the concept of a space‐time‐coding metasurface (STCM) for programmable range‐angle‐dependent energy auto‐scanning. The metasurface comprises phase‐reconfigurable unit cells with integrated PIN diodes on the bottom layer. These diodes are not visible in Figure 1 due to the top‐layer obscuring the underlying structural relationships. By dynamically tuning the bias voltage applied to each diode, the reflection phase of individual unit cells can be controlled. Crucially, as shown in the inset (Figure 1, left), identical periodic phase‐modulation waveforms are applied column‐wise but with progressive time delays (Δ*t*) between adjacent columns. This spatiotemporal coding scheme generate energy maxima (depicted as yellow S‐shaped‐curves in Figure 1) that autonomously trace evolving range‐angle trajectories over time, realizing continuous auto‐scanning without extra mechanical components.

**FIGURE 1:**
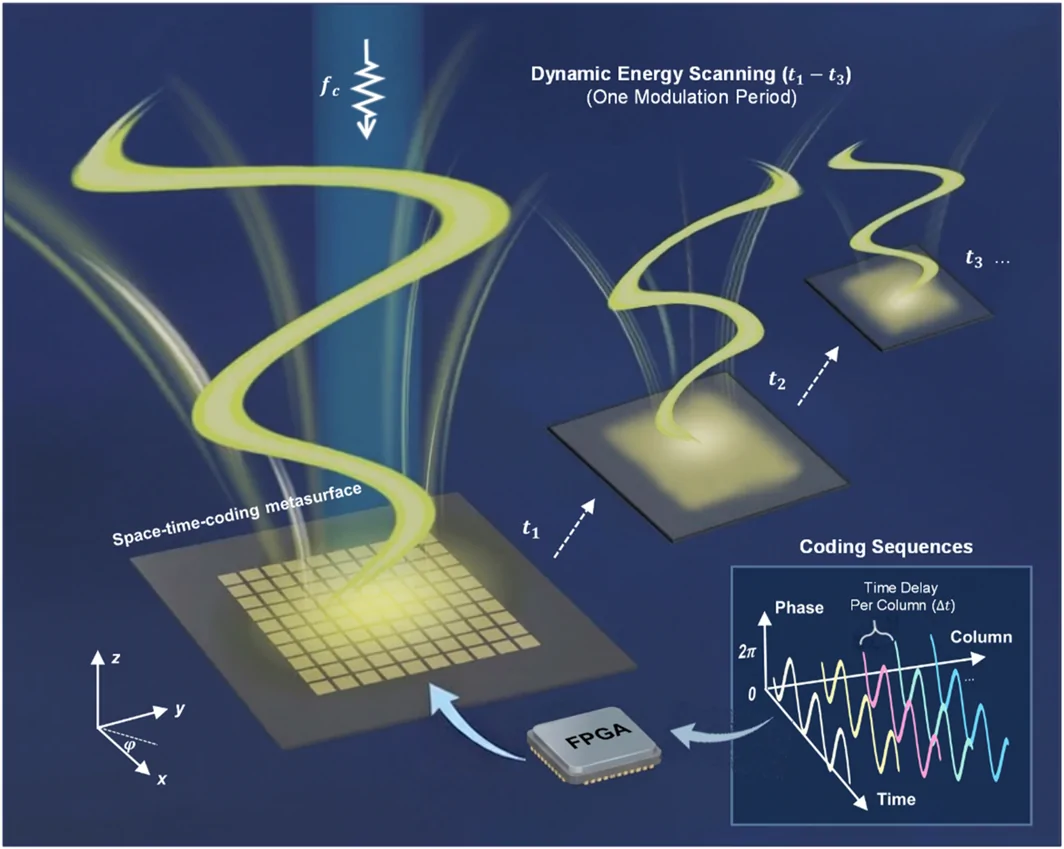
Conceptual illustration of spatial energy self‐scanning of a space‐time‐coding metasurface. By applying modulation signals with progressively increasing time delays to metasurface columns via FPGA, the electric field amplitude exhibits non‐uniform spatial distributions across angular and range while evolving temporally. In the illustrated example, snapshots at *t*_1_, *t*_2_, and *t*_3_ (three distinct time instances) demonstrate varying electromagnetic energy reflection patterns from the metasurface in one modulation period.

Assuming omnidirectional element patterns, previous studies have established that imposing a static reflection phase gradient along metasurface columns (Figure 2a) deflects the reflected beam to a specific angular direction (Figure 2b). The reflection phase states 00, 01, 10, and 11 in Figure 2a stands for 0°, 90°, 180°, and 270°, respectively, due to the 2‐bit resolution of the metasurface. As static modulation introduces no time‐varying components, the reflected wave consists exclusively of the fundamental frequency component (*f*_*c*_) matching the incident frequency. This spectral purity ensures that the total electric field's beam pattern corresponds precisely to the monochromatic radiation pattern at *f*_*c*_, which maintains a stationary spatial distribution (Figure 2e). To introduce spatiotemporal dynamics, identical time‐varying modulation sequences are applied across metasurface columns, with a progressive time delay distribution such that the modulation signal for the *n*th column is time‐shifted by (*n*−1)Δ*t* relative to the first column. This results in a shift in reflected frequency and a phase gradient along the spatial dimension of the metasurface. When temporal phase modulation operates exhibits linear variation at frequency *f*_*m*_ (Figure 2c), the system predominantly generates a single Doppler‐shifted harmonic at *f*_*c*_ + *f*_*m*_ (Figure 2d). The total reflected electric field distribution remains time‐invariant since the progressive time delays of a linear phase variation function maintain a constant inter‐column phase difference 2π*f*_*m*_Δ*t* over time. This stable phase gradient preserves fixed spatial coherence across the aperture. The reflected energy distribution therefore remains locked in a single direction without temporal evolution or range dependence as demonstrated in Figure 2e.

**FIGURE 2:**
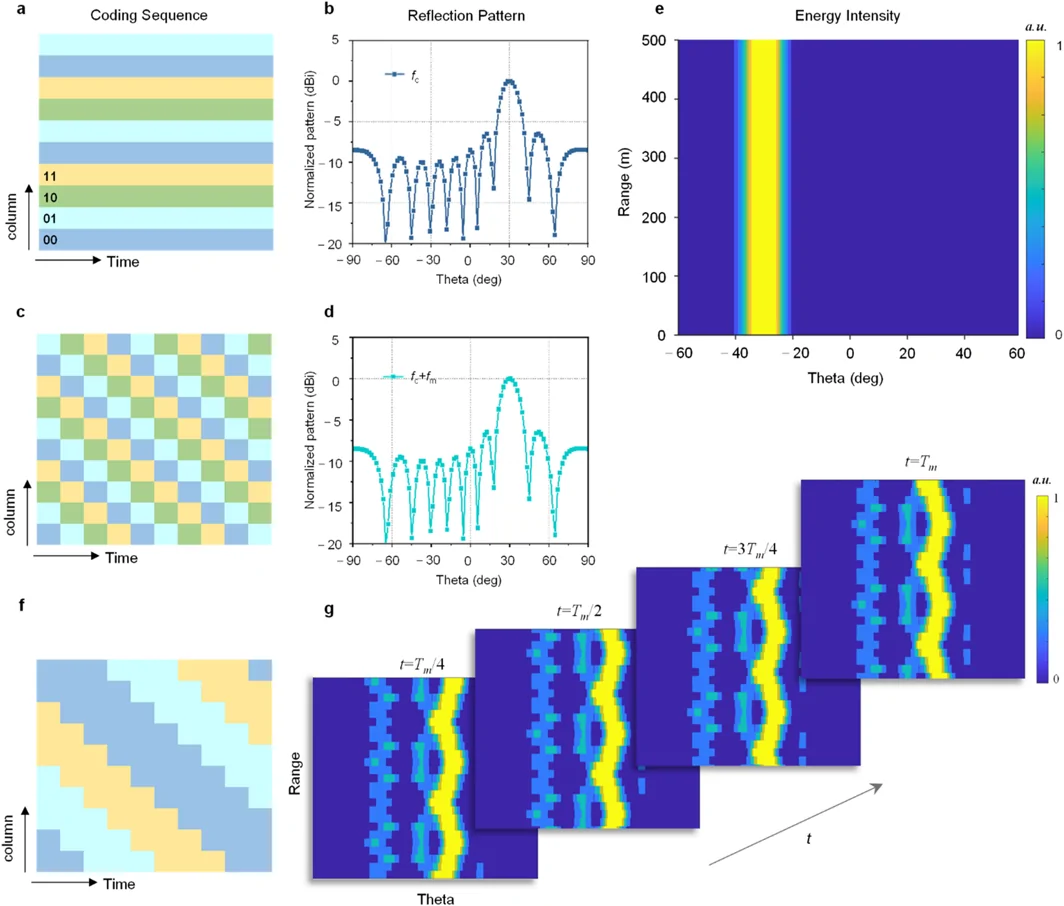
Coding sequences, reflection patterns and energy intensity distribution for three cases. (a) Coding sequences of time‐invariant case. (b) Reflection pattern of time‐invariant case. (c) Coding sequences of linearly time‐modulated case. (d) Reflection pattern of linearly time‐modulated case. (e) Energy intensity distribution in *φ* = 90° plane of metasurface under coding sequence in a or c. (f) Coding sequences of nonlinearly time‐modulated case. (g) Energy intensity distribution of metasurface in *φ* = 90° plane under coding sequence in f within one modulation period with interval.

In this work, a nonlinear modulation sequence is implemented while maintaining progressive time delays between columns (Figure 2f). Neglecting losses during the transmission process, suppose that the incident wave propagating along – *z* direction takes the form of

(1) $$\begin{array}{c} E^{i}\left(z,t\right)={E_{0}e}^{j2{\pi f}_{c}t}e^{jk_{0}z} \end{array},$$

where $E_{0}$ represents the magnitude of incident electric field, and $k_{0}$ represents the propagation constant at $f_{c}$. The total reflected electric field can be expressed as

(2) $$\begin{array}{c} E^{r}\left(t,r,\theta ,\varphi \right)=\sum_{n=1}^{N}E_{n}^{r}\left(t,r,\theta ,\varphi \right), \end{array}$$

$E_{n}^{r}$ represents the reflected electric field of column *n* of the metasurface, *N* is the total metasurface column number, *t* represents observation time, *r* represents the propagation distance of the reflected wave, and *θ* and *φ* are the elevation and azimuth angles. When the metasurface is placed at *z* = 0 plane by assuming that $f_{m}$ is much smaller than $f_{c}$, and the unit cell exhibits an omnidirectional pattern, $E_{n}^{r}$ in *φ* = 90° plane can be written as

(3) $$E_{n}^{r}\left(t,r,\theta \right)=E_{0}e^{j2\pi f_{c}(t‐\frac{r}{c})}e^{j\Phi_{n}(t‐\frac{r}{c})}e^{jk_{0}d(n‐1)sin\theta }$$

where $\phi_{n}$ is the time‐varying reflection phase of column *n*, and d denotes the side length of the metasurface unit. The independent variable t is taken as $t-\frac{r}{c}$ to account for the retarded phase factor when calculating the radiated field, which is particularly significant as it establishes a connection between the variation of modulation signal in time domain and the distribution of the electric field along the range dimension. Supposing that the nonlinear modulation sequence be expressed as

(4) $$\begin{array}{c} \phi_{n}\left(t\right)=\phi \left(t+\left(n-1\right)\Delta t\right). \end{array}$$

The total electric field distribution can be written as

(5) $$E^{r}\left(t,r,\theta \right)=E_{0}e^{j2\pi f_{c}(t-\frac{r}{c})\;}\sum_{n=1}^{N}e^{j\phi (t-\frac{r}{c}+\left(n-1\right)\Delta t)}e^{jk_{0}d\left(n-1\right)\sin\;\theta }.$$

It is evident that the absolute value of total reflection field is dependent on range, angle, and time. As shown in Figure 2g, the resulting field distribution exhibits dynamic spatiotemporal evolution. Due to the periodicity of $\phi_{n}\left(t\right)$ with fundamental frequency *f*_*m*_, the variation period is *T*_*m*_ = 1/*f*_*m*_. From a spectral perspective, the nonlinear modulation decomposes the reflected field into multiple harmonic frequencies *f*_*c*_ ± *kf*_*m*_. The complex reflectivity for the *n*th column at the *k*th harmonic is $a_{n}^{k}=a_{k}e^{j2\pi kf_{m}\left(n-1\right)\Delta t}$ according to Fourier transform, where $a_{k}$ is governed by the Fourier spectrum of $\phi_{n}\left(t\right)$. Therefore, the electric field of the *k*th‐order harmonic wave is

(6)

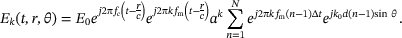




Equation (6) shows that each harmonic component maintains a static beam pattern. The *k*‐th harmonic beam is directed toward a fixed angular direction $\theta_{k}=\arcsin(-\frac{\;2\pi kf_{m}\Delta t}{k_{0}d})$ in the *φ* = 90° plane, exhibiting no temporal variation or range dependence. The dynamic evolution of the total electromagnetic field emerges from the interference between harmonic beams at distinct frequencies.

### 2.2 Space‐Time Coding Synthesis for Energy Trajectory Design

This section details the methodologies for designing energy scanning trajectories using the proposed modulation scheme. The first methodology is the phase‐gradient‐based trajectory design approach. As established previously, the range‐dependent energy variation fundamentally arises from retarded phase effects in the radiated field calculation. In an idealized scenario where the phase profile can be arbitrarily controlled at each instant, designing the desired spatiotemporal energy distribution directly equates to designing the main beam direction at each specific time instant, which is functionally equivalent to designing the phase gradient of the metasurface at every moment.

Precise direct trajectory control proves challenging (see Supporting Information S1: Note 1). Given the practical constraint *N*Δ*t* ≪ *T*_*m*_, and approximately constant phase slope of ϕ(t) over *N*Δ*t*, the first‐order Taylor expansion at the φ=90° plane yields the incremental phase relationship as follows:

(7) $$\begin{array}{c} \phi \left(t+\Delta t\right)-\phi \left(t\right)=\frac{d\phi \left(t\right)}{dt}\Delta t+o\left(\Delta t^{2}\right) \end{array}.$$

Maximizing |*E*| requires phase matching between adjacent elements. According to Equations (5) and (7), this necessitates the following:

(8) $$\begin{array}{c} \frac{d\phi \left(t\right)}{dt}\Delta t+k_{0}d\;\sin\;\theta_{\text{scan}}=0 \end{array}.$$

Solving for the instantaneous scanning angle gives the following:

(9) $$\begin{array}{c} \sin\;\theta_{\text{scan}}\left(t\right)=-\frac{\Delta t}{k_{0}d}\frac{d\phi \left(t\right)}{dt}. \end{array}$$

Thus, the instantaneous main lobe direction $\theta_{\text{scan}}\left(t\right)$ is governed by the product of the wavefront progression velocity $\frac{\Delta t}{k_{0}d}$ and the phase slope $\frac{d\phi \left(t\right)}{dt}$. This linear dependence $\sin\;\theta_{\text{scan}}\left(t\right)\propto \frac{d\phi \left(t\right)}{dt}$ permits arbitrary trajectory engineering through temporal shaping of ϕ(t), establishing the phase sequence as the sole design degree of freedom for spatiotemporal control.

Figure 3 demonstrates the application of this methodology for two distinct scanning patterns: linear and S‐shaped‐curve scanning. Specifically, Figure 3a presents the designed continuous phase sequence ϕ(t) for linear scanning, which follows a trigonometric function, while Figure 3d shows the corresponding sequence for S‐curve scanning based on a quadratic function. Subject to hardware constraints, ϕ(t) is discretized into 1‐μs time steps with 2‐bit phase resolution. The simulated energy intensity distributions after this discretization are shown in Figure 3b,e, respectively. Figure 3c,f plot the angle corresponding to the energy maximum as a function of range under the two modulation schemes. The results demonstrate that the 2‐bit quantized waveforms achieve trajectory accuracy comparable to that of the ideal continuous ϕ(t), with an angle error of less than 5°. This confirms the robustness of the scheme to hardware simplifications, maintaining high fidelity despite severely limited phase resolution. These two examples presented above correspond to single‐beam scanning trajectories. In principle, however, phase‐gradient‐based trajectory design approach can be extended to multi‐beam designs by superimposing the coding sequences required for each individual beam trajectory.

**FIGURE 3:**
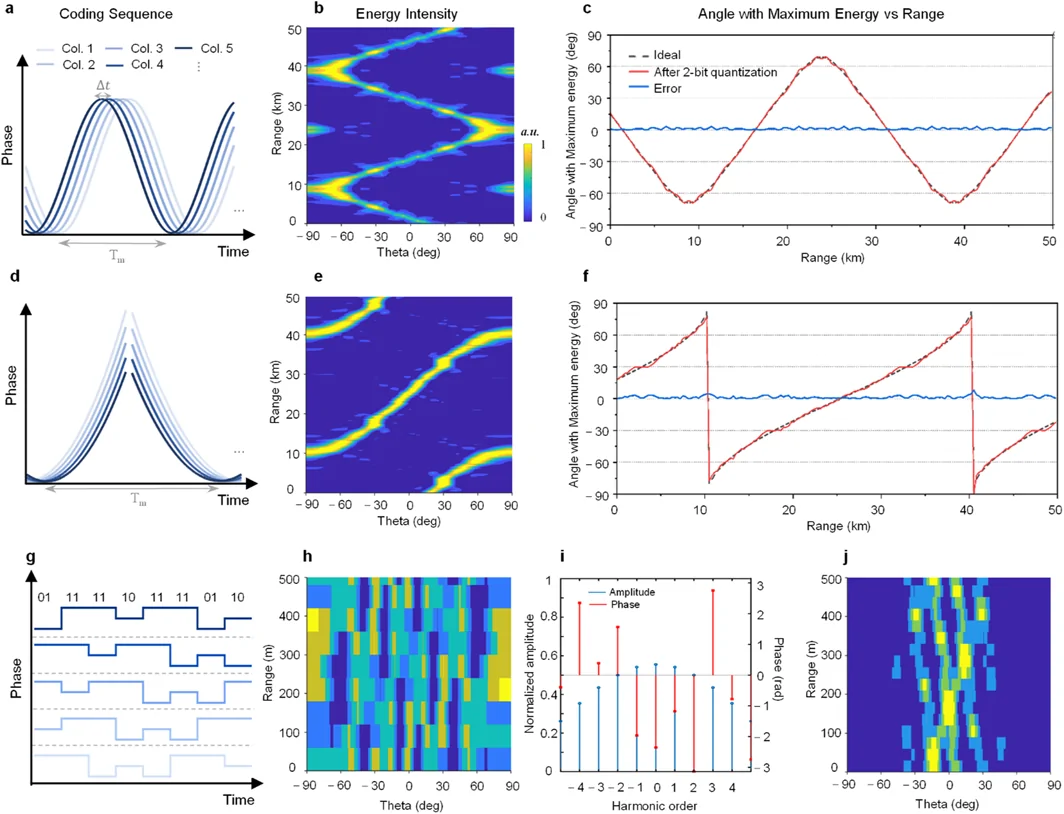
Implementation of energy trajectory design for STCMA. (a) Modulation sequence $\phi \left(t\right)=K_{1}\;\sin\left(2\pi f_{m}t\right)$, where $K_{1}$ is a constant. (b) The distribution of energy reflected by metasurface under coding sequence in a. (c) Main beam angle versus modulation parameter before and after 2‐bit quantization of the metasurface modulated using coding sequence in a, and the corresponding pointing error. (d) Modulation sequence ${\phi \left(t\right)=K}_{2}t^{2}$, where $K_{2}$ is a constant. The observed periodicity is not inherent but is artificially imposed by applying the sequence in a repetitive manner. (e) The distribution of energy reflected by metasurface under coding sequence in d. (f) Main beam angle versus modulation parameter before and after 2‐bit quantization of the metasurface modulated using coding sequence in d, and the corresponding pointing error. (g) Modulation sequence “01‐11‐11‐10‐11‐11‐01‐10” for harmonic spectrum engineering across space. (h) c under coding sequence in g. (i) The spectrum in one bandwidth of coding sequence in g. (j) The distribution of energy reflected by metasurface under coding sequence in g, but with time delay reduced to $\frac{1}{2\text{BW}}$.

The above derivation assumes *N*Δ*t* ≪ *T*_*m*_. Nevertheless, when this condition cannot be met, a fundamentally different design strategy must be adopted. Instead of directly designing the instantaneous main beam direction through the phase gradient based on the approach above, we treat the desired total energy distribution as the incoherent sum of multiple harmonic beams, a strategy we refer to as harmonic spectrum engineering. This strategy exploits the principle that the directionality, relative intensities, and phase relationships among harmonic beams collectively govern the spatial concentration of energy (Supporting Information S1: Note 2). To achieve a more uniform distribution of reflected energy across different angles, an exhaustive search is conducted across the coding space to minimize harmonic gain disparities. This optimization produced the coding sequence in Figure 3g and the corresponding energy distribution in Figure 3h. The resulting harmonic spectrum (Figure 3i) shows well‐balanced amplitudes, enabling broad and uniform coverage across angular space.

In addition to the selection of ϕ(t), the progression velocity $\frac{\Delta t}{k_{0}d}$ is critically important as it governs the fundamental angular scaling limits of the system. Specifically, for full‐angle scanning under element spacing *d* = *λ*_*c*_/2, Δ*t* must exceed 1/BW, where BW is the 90% energy bandwidth of the modulation sequence bandwidth. This condition Δ*t* ≥ 1/BW provides a sufficient guarantee that the full‐angle scanning requirement is met. This theoretical constraint is validated in Figure 3h,j by simulation, which compare energy distributions using the same coding sequence under different Δ*t* values. Figure 3h demonstrates optimal performance at Δ*t* = 1/BW, whereas reducing Δ*t* to 0.5/BW (Figure 3j) results in a substantial contraction of the scanning range due to violation of this bandwidth constraint.

### 2.3 Experiment Verification

To validate the proposed concepts and experimentally verify the spatial energy self‐scanning capability, a 10 × 10 2‐bit reconfigurable metasurface prototype is designed, fabricated, and tested in an indoor environment. The prototype, with overall dimensions of 285 × 285 × 3.5 mm, is fabricated as a multi‐layer structure [50]. This stack consists of three metal layers separated by two F4B substrate layers (*ε*_*r*_ = 2.55, tan*δ* = 0.001) with thicknesses of 3 and 0.5 mm, respectively. The unit cell has a side length of 18.5 mm, corresponding to approximately half a wavelength at 5 GHz within the substrate. As illustrated in Figure 4a, the top metal layer comprises an array of square patches that receive and radiate electromagnetic waves. The intermediate metal layer serves as a common ground for the structure. The core reconfigurability is enabled by a phase‐shifter integrated into the bottom layer of each unit cell, which is detailed in the zoom‐in view of Figure 4a. The phase‐shifter is actuated by three PIN diodes (MADP000907; *R* = 5.2 Ω, *L* = 0.1 nH, and *C* = 0.02 pF). An external FPGA controls the ON/OFF states of these diodes via a pair of bias lines. By digitally switching the diode states, each unit cell can be programmed to one of four distinct phase states with a 90° step resolution (see Supporting Information S1: Note 4). This precise phase control facilitates the necessary wavefront manipulation to achieve the desired self‐scanning functionality.

**FIGURE 4:**
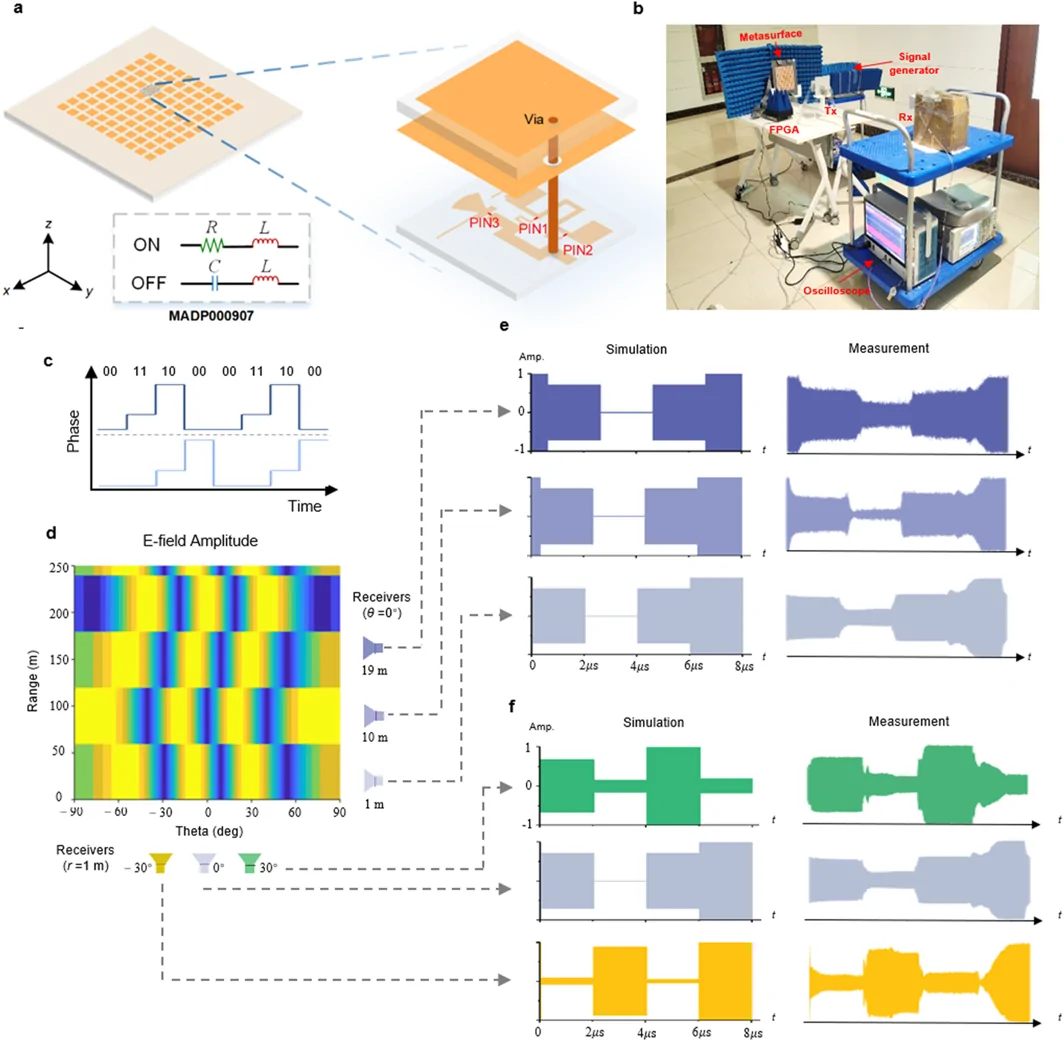
Prototype design, testing, and characterization. (a) Schematic of the realized space‐time‐coding metasurface prototype. (b) Measurement setup in an indoor environment. (c) Modulation sequence “00‐01‐10‐11” for experiment. (d) Electric field distribution reflected at *φ* = 90° plane under coding sequence “00‐01‐11‐00”. (e) Simulated versus measured real parts of normalized electric field waveforms over a single temporal period at *φ* = 90° for ranges of 1 m, 10 m, and 19 m. (f) Simulated versus measured real parts of normalized electric field waveforms over a single temporal period at *r* = 1 m and azimuth angle of 30°, 0°, and 30°.

The experiment was conducted in spacious corridor, with its setup illustrated in Figure 4b. Aside from the STCM, the transmission part of measurement setup mainly composed of an FPGA platform (ALTERA Cyclone IV), a microwave signal generator (Keysight E8257D), and a linearly polarized horn. The receiving part consists of a horn antenna and an oscilloscope (TEKTRONIX MSO64B). The metasurface is mounted on a 3D‐printed base that can be manually rotated. To maintain a consistent normal incidence angle during testing, the transmitting horn antenna is fixed to this same base via a crossbar. The base is marked with graduations to accurately measure the rotation angle. Rotating the graduated base in the horizontal plane simultaneously turns both the metasurface and the transmitter, which is functionally equivalent to altering the angle of wave reception. Meanwhile, the receiving terminal is set on a lab cart, allowing the reception range to be varied by moving the cart. To minimize the impact of the testing environment on the results, all highly reflective objects in the vicinity of the metasurface are covered with microwave‐absorbing material.

To ensure experimental feasibility, the metasurface's coding pattern is simplified by assigning identical sequences to every five columns, resulting in two unique sets. Each set is programmed with a 4‐state periodic sequence “00‐01‐11‐00”, modulated at a frequency $f_{m}$ of 1.25 MHz and time delay Δ*t* of 0.2 μs. This modulation corresponds to a diode switching frequency of 5 MHz and a modulation period T of 0.8 μs. This configuration satisfies the full‐angle scanning requirement inherent to the harmonic spectrum engineering approach. The resulting electric field distribution in the *φ* = 90° plane is shown in Figure 4d.

Figure 4e presents simulated and measured normalized electric field waveforms over a single period at a 0° receiving angle for distances of 1, 10, and 19 m. To accurately capture the time‐varying periodic received field, a synchronous acquisition mechanism is employed, wherein each measurement was triggered at a fixed time point of the modulation period. This approach enabled consistent extraction of E‐field information while eliminating temporal misalignment across comparisons (see Methods for details). The results show good agreement between simulation and experiment, confirming the validity of the testing approach and the energy‐scanning theory. Minor amplitude distortions are attributed to the finite response time of the diodes. A clear temporal delay is observed with increasing distance, validating the metasurface's range‐scanning capability. Figure 4f shows the corresponding waveforms at a fixed distance of 1 m for receiving angles of −30°, 0°, and 30°. The normalized measurements align well with simulations and clearly exhibit the anticipated angular‐scanning behavior. Together, these results demonstrate distinct dependencies on both angle and range, confirming the STCM's ability to perform two‐dimensional energy scanning across the horizontal plane.

### 2.4 New‐Architecture Wireless Target Positioning System

The energy self‐scanning capability of space‐time coding metasurfaces generates unique time‐varying energy distribution patterns with an intrinsic angle‐range correlation. This property allows single‐channel receiving systems to achieve high‐accuracy single‐target localization using super‐resolution algorithms such as MUSIC [51, 52]. Since the energy distribution naturally depends on both range and angle, and no two distinct points produce identical temporal waveforms, MUSIC directly yields decoupled range and angle estimates without requiring additional hardware or algorithmic modifications. This advancement enables decoupled and high‐accuracy estimation of both azimuth angle and distance (see Supporting Information S1: Note 5 for detailed analysis), representing a critical step toward high‐precision unambiguous positioning. In contrast, for conventional FDA, decoupling typically requires non‐uniform frequency increments or non‐uniform element spacing.

To validate this concept, a novel range‐angle estimation system is proposed based on a space‐time coding metasurface (STCM). In a conventional bi‐static radar setup, signals emitted by the transmitter are reflected by targets and then captured by the receiver. To reduce hardware complexity while preserving functional equivalence, a simplified experimental setup is implemented in which the receiving horn is placed directly at the target location, as shown in Figure 4b. In this configuration, the received signal is functionally equivalent to the reflected signal by the target point in a conventional bistatic scenario. Although this setup replaces the typical round‐trip propagation with a one‐way path, the simplification is justified because the temporal properties of the received signal retain a direct correlation with the target's spatial position. Specifically, the MUSIC algorithm matches the received waveform with a theoretical waveform derived from the same one‐way distance *r*, so the spatial spectrum peak directly yields the true target position. Thus, the geometric information essential for accurate localization is preserved.

The experimental design retains the modulation scheme validated in prior verification tests to strike a balance between positioning performance and practical implementation constraints. For parameter estimation, the multiple signal classification (MUSIC) algorithm is employed. Simulated results derived from the processed ideal data are presented in the form of spatial spectra (Figure 5a), which clearly exhibits sharp energy foci consistent with theoretical expectations, thereby confirming the algorithm's efficacy under idealized simulation conditions. The system's performance is then tested by testing five point targets individually, each placed at a distinct location. All measurements are conducted with a single target present at a time, as the current system is designed for single‐target localization. Since the PIN diode (MADP000907) used in our system has a typical switching time of 2–3 ns, to compensate for systematic timing offsets caused by diode switching, an additional reference‐based calibration procedure is adopted (see Supporting Information S1: Note 6 for details). The measured signal‐to‐noise ratio (SNR) ranged from approximately 22 to 44 dB depending on the target location (see Supporting Information S1: Note 7 for details), ensuring reliable MUSIC processing. Since that the metasurface elements exhibit a non‐isotropic pattern, resulting in low energy at large angles, the angle estimation range in this work is confined to ± 20° to ensure sufficient signal‐to‐noise ratio at the receiver. As shown in Figure 5b, in realistic circumstance, the estimated locations show excellent agreement with the preset target positions. Error analysis (Figure 5c,d) shows maximum errors of 3° in angle and 0.2 m in range, with slight deviations mainly due to target placement accuracy. These results strongly affirm the effectiveness of the positioning algorithm and overall system reliability.

**FIGURE 5:**
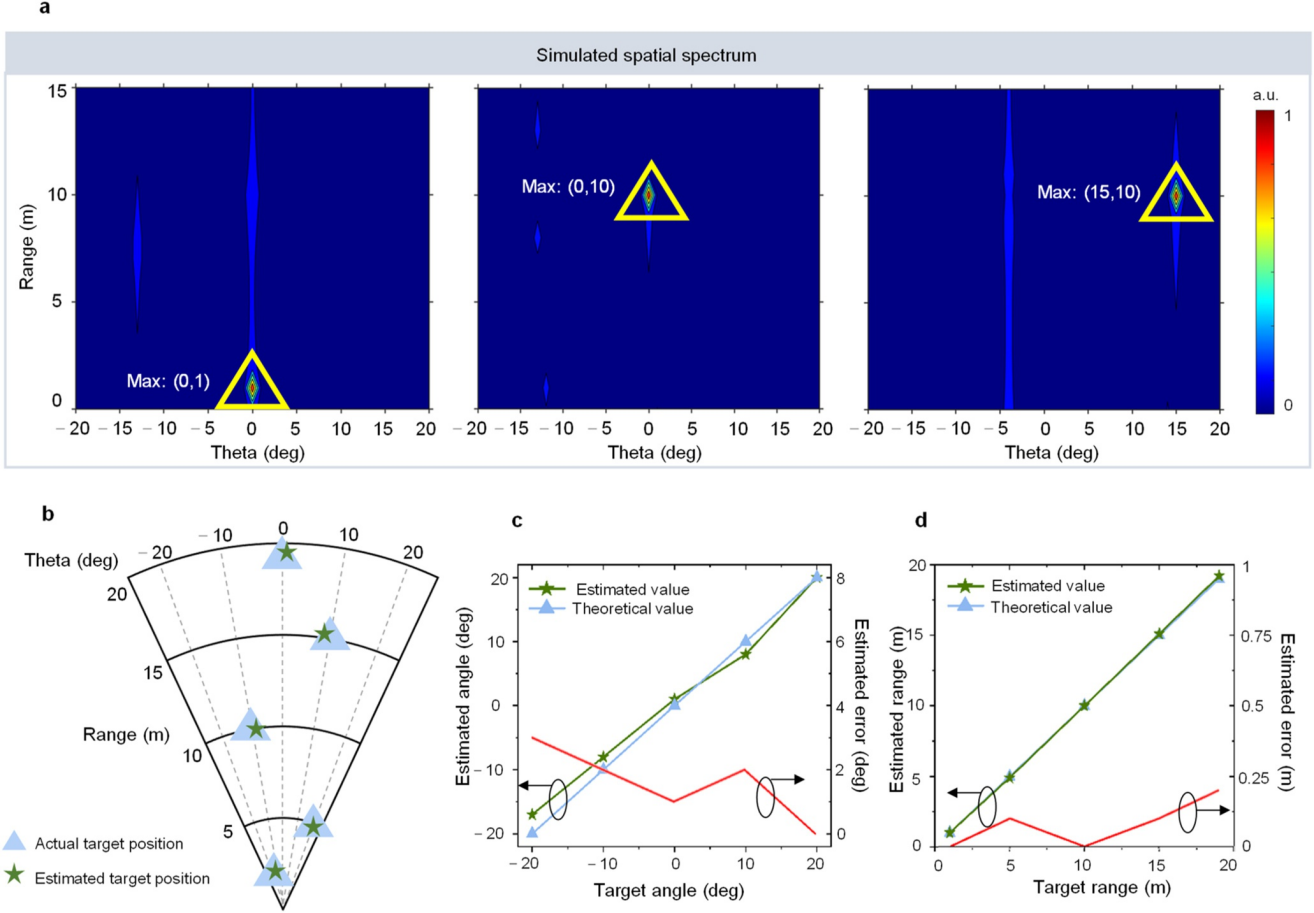
Simulation and measured results for wireless positioning system. (a) Simulated spatial spectrum for targets located at (0°, 1 m), (0°, 10 m), and (15°, 1 m). The regions of maximum spectral intensity correspond to the estimated target positions. (b) Comparison of estimated target positions with actual target positions. (c) Angular error analysis of target positioning for varying target angles. (d) Range error analysis of target positioning for varying target ranges.

## 3 Discussion

In conclusion, this study demonstrates a paradigm shift in electromagnetic control through spatiotemporal coding metasurfaces enabled by progressive time‐delay modulation. By applying nonlinear time‐varying modulation signals with incremental temporal delay across metasurface columns, the metasurface can achieve energy self‐scanning without the need of complicated feeding network, nor asynchronous modulated signal. By judiciously designing coding sequences, the desired energy trajectory can be effectively achieved. As proof of concept, energy intensity distributions in φ=90° plane is simulated, while waveforms across varying angles and ranges is simulated and tested. A metasurface prototype is fabricated for experimental verification. The measured results show remarkable agreement with numerical simulations. Furthermore, a positioning system based on the proposed STCM is constructed, wherein the MUSIC algorithm achieved super‐resolution positioning for five targets distributed at distinct angular and range coordinates. This experimental‐theoretical consistency confirms the effectiveness of spatiotemporal coding strategy proposed by this paper in practical implementations.

By comparison with array based scanning systems, the STCM approach offers significantly enhanced convenience and flexibility, boasting advantages such as simplified architectures, versatile modulation capabilities, reduced fabrication costs, improved operational efficiency, and multifunctional performance. Moreover, the proposed progressive time‐delay strategy overcomes the inherent angle‐range coupling limitation of frequency‐diverse architectures, enabling decoupled energy scanning through dynamic wavefront shift mechanisms.

This work fundamentally redefines the operational paradigm of electromagnetic control by establishing a direct correspondence between spatiotemporal coding sequences and energy distribution patterns. Beyond positioning applications, the technology unlocks transformative potential for secure directional wireless communication and adaptive energy transfer systems, where dynamic energy redistribution capabilities become paramount. Future investigations should focus on developing machine‐learning‐optimized nonlinear coding strategies and addressing the challenges associated with quasi‐static electric field distribution management. Nevertheless, by evolving STCMs from conventional wave parameter modulators into spatiotemporal energy directors, this platform establishes a foundation for electromagnetic environments capable of cognitive‐level adaptability—a critical advancement toward intelligent electromagnetic ecosystems.

## 4 Methods

### 4.1 Simulations of the Metasurface

In this study, the metasurface is designed using CST Microwave Studio 2023. The reflection amplitude and phase of the unit cells are optimized by adjusting their geometry and dimensions, achieving a reflection loss of less than 2 dB and a phase difference between adjacent coding states of 90° ± 10°. Unit cell simulations are performed using the Frequency Domain Solver with unit cell boundary conditions. Subsequently, a 10 × 10 array including all bias lines is simulated under plane wave excitation using the Time Domain Solver. Wave deflection capability under normal incidence is simulated by implementing a linear phase gradient across the array. The energy distribution and received signals are simulated in MATLAB 2023a under the ideal assumption of a perfect metasurface reflection phase, without incorporating phase errors or return loss.

### 4.2 Experimental Measurement Setup

The metasurface is fabricated using standard printed circuit board (PCB) technology, and its supporting frame is manufactured via 3D printing. The signal generator transmitted a continuous sine wave at a fixed power level of 25 dBm. To capture the time‐varying electric field characteristics, in the experiment, an FPGA is used to generate both a reference signal and the control signals for the metasurface. The reference signal is a square wave with a 50% duty cycle and a period same as that of modulation signal. Both the reference signal and the output from the receiving horn are connected to an oscilloscope for synchronized acquisition. The oscilloscope is triggered on the rising edge of the reference signal to ensure stable and coherent signal recording. Since the electromagnetic distribution is equivalent at identical time instances across different periods, we are able to measure far‐field signals at different spatial points over successive cycles. The electric field signal collected between two consecutive rising edges of the reference signal is taken as one complete dataset for analysis. The oscilloscope sampling rate is set to 50 Gpts/s. Data acquired by the oscilloscope are transferred to a laptop via Ethernet for offline processing and further analysis.

### 4.3 High‐Resolution Parameter Estimation via MUSIC

The multiple signal classification (MUSIC) algorithm is employed to achieve high‐resolution joint range and angle estimation. All simulations are performed using MATLAB R2023a. The actual reflection amplitude and phase of the metasurface under each coding state were measured and directly incorporated into the MUSIC algorithm to calibrate the system model. The received electric field data are first normalized to their maximum value prior to processing to ensure numerical stability. The spatial covariance matrix is estimated using 1000 snapshots to ensure statistical reliability. The two‐dimensional MUSIC spatial spectra is computed over a uniformly discretized parameter space to facilitate target localization. The angular domain is sampled from −30° to 30° with an increment of 1°, while the range dimension is evaluated from 1 to 20 m with a step size of 0.1 m. This dense sampling strategy enabled unambiguous and precise retrieval of target parameters through peak detection in the resulting range‐angle spectrum.

## Author Contributions

**Yuzhen Chen:** conceptualization, methodology, writing – original draft, software, data curation, investigation, validation, visualization. **Xiaoyi Wang:** writing – review and editing, investigation, methodology, conceptualization, project administration. **Guo‐Min Yang:** project administration, resources, supervision, writing – review and editing, funding acquisition. **Ya‐Qiu Jin:** resources, supervision.

## Conflicts of Interest

The authors declare no conflicts of interest.

## Code Availability

The custom computer codes used in this study are available from the corresponding authors on request.

## Supporting information


Supporting Information S1

## Data Availability

The authors declare that all relevant data are available in the paper and its Supporting Information S1 files, or from the corresponding author on request.
