# Supplementary material for: Space‐Time‐Coding Metasurfaces Enabling Programmable Range‐Angle‐Dependent Energy Auto‐Scanning
Source: Nanophotonics. 2026 Jul 16;15(14):e70204. doi: 10.1002/nap2.70204 (PMC13642473; doi:10.1002/nap2.70204)
Supplement: Supplementary file 1 [file NAP2-15-e70204-s001.docx]

Supplementary Information for

**Space-Time-Coding Metasurfaces Enabling Programmable** **range-angle-dependent energy Auto-Scanning**

**This supplementary information includes:**

Supplementary Note 1. The challenge of precise energy trajectory control

Supplementary Note 2. The relationship between harmonics and energy spatial distribution

Supplementary Note 3. Relationship between Δ*t* and energy angular coverage

Supplementary Note 4. Design of the metasurface

Supplementary Note 5. The principle of decoupled angle and range estimation

Supplementary Note 6. Reference-based calibration for range estimation

Supplementary Note 7. Signal-to-Noise Ratio Analysis

**Supplementary Note 1. The challenge of precise energy trajectory control**

According to Equation (5) in the main text, maximizing the total reflected electric field requires phase matching condition

$$\begin{aligned} \phi\left( t+\left( n-1 \right)\Delta t \right)+k_{0}d\left( n-1 \right)\sin\theta_{target}=0\#\text{(S1)} \end{aligned}$$

to be satisfied for all *n*. Under this condition,

$$\begin{aligned} \phi\left( t+\left( n-1 \right)\Delta t \right)=-k_{0}d\left( n-1 \right)\sin\theta_{target}\left( t \right).\#\text{(S2)} \end{aligned}$$

It is noted that this condition must hold simultaneously for all *n*. Assuming the existence of a function $\phi\left( t \right)$ that satisfies the above relation. The left-hand side of the $\text{(S2)}$ is a function of *t* + (*n*−1) Δ*t*, while the right-hand side is a function of *t* and is proportional to (*n*−1). This implies that the equality cannot hold for all *n* unless $\phi\left( t+\left( n-1 \right)\Delta t \right)$ is also proportional to (*n*−1). Such proportionality would require that the $\phi\left( t \right)$ is a linear function of time. If $\phi\left( t \right)$ is a linear function of time, consequently,$\theta_{target}$(t) would also be constant—a condition that contradicts the requirement for energy scanning. Therefore, it is not possible to precisely design the modulation sequence using conventional phase matching methods.

**Supplementary Note 2. The relationship between harmonics and energy spatial distribution**

**Fig. S1 Coding sequences, spectrum, harmonic radiation patterns, and spatial electric field distribution under four scenarios. a** The time delay of modulation signals controlling adjacent metasurface columns is set to 2*tₘ*. **b** The number of columns in the metasurface is increased to 12. **c** The metasurface adopts coding sequence of “10-10-10-00-00-00-00-00”. **d** The metasurface employs a 2-bit coding scheme. In the left schematic diagram of the unit-cell states, green blocks correspond to the ‘01’ state (90° reflection phase) and purple blocks represent the ‘11’ state (270° reflection phase).

As discussed, the total electrical field distribution in space constitutes the sum of individual harmonic components, and only nonlinear modulation signals enable dynamic electromagnetic energy distribution in space. Fig. S1 analyzes the relationships between modulation parameters, harmonic characteristics and spatial energy distribution. As shown in Fig. S1a, increasing the time delay between metasurface columns to 2*t_m_* enhances the equivalent phase gradient at each harmonic frequency, enlarging the main-beam angular difference between adjacent harmonics. This results in fewer distinguishable beam directions and the emergence of null regions in the electric field distribution.

Fig. S1b demonstrates the number of metasurface columns determines the beamwidth of harmonic patterns. Increasing column count narrows the main beam, creating irreducible coverage gaps between discrete beams that cannot be compensated by inherent beams. Fig. S1c reveals that the coding scheme of modulation sequences directly impacts harmonic intensities. Under the coding configuration shown in Fig. S1c, the ±1st-order harmonics exhibit significantly higher gains than other harmonics, thereby concentrating the radiated energy within their main lobes.

However, as shown in Fig. S1, the 1-bit metasurface exhibits mirror symmetry electric field distribution about the *θ*=0∘ plane, introducing inherent angular ambiguity in localization that requires extra spectral analysis for resolution. This angular ambiguity originates in the spectral characteristics of 1-bit coding sequences, where their even-symmetric amplitude spectra and odd-symmetric phase spectra generate congruent field amplitude distributions at symmetrical angular positions. Consequently, higher-bit coding is essential for breaking this symmetry in positioning applications.

Fig. S1d presents the spectral characteristics of the 2-bit coding scheme, where the asymmetric phase distribution evident in the frequency domain facilitates non-symmetric spatial field patterns, effectively overcoming the symmetry limitations inherent in 1-bit coding, providing an ideal ambiguity-free electric field distribution for positioning systems.

**Supplementary Note 3. Relationship between Δ*t* and energy angular coverage**

Assuming the metasurface has a total of *N* columns, and the signal reflected by each column is denoted as $E_{n}^{r}(t)$, which has a frequency bandwidth BW. To achieve full spatial energy coverage (i.e., the ability to steer the reflected beam to any angle between $-$90° and +90°), the phase shift difference across the metasurface must be sufficient to cover the entire range of required phase gradients.

The relationship between the time delay $\Delta t$ applied to the signal and the resulting phase shift $\Delta\varphi$ is frequency-dependent:

$$\begin{aligned} \Delta\varphi\left( f \right)=2\pi f\Delta t.\#\text{(S3)} \end{aligned}$$

Therefore, for a signal with bandwidth BW, the difference in phase shift between the highest and lowest frequencies is:

$$\begin{aligned} \Delta\varphi_{\max}-\Delta\varphi_{\min}=2\pi\mathrm{BW}\Delta t.\#\text{(S4)} \end{aligned}$$

This range of phase shifts directly corresponds to a range of beam steering angles. To ensure the beam can be steered to both extremes of the hemisphere (i.e., －90° and +90°), this phase difference must be large enough. The required phase difference to steer a beam to an angle $\theta$ is also given by the standard array theory formula for an element spacing *d*:

$$\begin{aligned} \Delta\varphi=(2\pi/\lambda)d\sin\theta.\#\text{(S5)} \end{aligned}$$

Since the bandwidth is much smaller than the center frequency, approximating$\lambda_{\max}$=$\lambda_{min}=\lambda_{0}$, the phase difference needed for extreme angles (*θ*=±90∘) is approximately:

$$\begin{aligned} \Delta\varphi_{\max}-\Delta\varphi_{\min}=(2\pi/\lambda_{0})d\left[ \sin\left( 90^{\circ} \right)-\sin\left( -90^{\circ} \right) \right]=(4\pi/\lambda_{0})d.\#\text{(S6)} \end{aligned}$$

Equating (S4) and (S6) for the required phase difference yields:

$$\begin{aligned} 2\pi\mathrm{BW}\Delta t\geq(4\pi/\lambda_{0})d.\#\text{(S7)} \end{aligned}$$

For the common array element spacing of$d=\frac{\lambda_{0}}{2}$ (chosen to avoid grating lobes), this condition simplifies to:

$$\begin{aligned} \Delta t\geq\frac{1}{\mathrm{BW}}.\#\text{(S8)} \end{aligned}$$

Therefore, to achieve full spatial energy coverage, the time delay $\Delta t$ must be greater than or equal to the reciprocal of the signal’s bandwidth $\frac{1}{\mathrm{BW}}$. This ensures the metasurface can generate the necessary range of phase shifts across the entire signal bandwidth to steer the beam to any angle.

**Supplementary Note 4. Design of the metasurface**

**Fig. S2**. **Bottom-layer structure of the metasurface unit cell**

**Fig. S3.** **Simulation results of the proposed 2-bit unit cell. a** Reflection amplitude of 2-bit unit cell for *x*-polarized incidence and *y*-polarized incidence. **b** Reflection phase of 2-bit unit cell for *x*-polarized incidence and y-polarized incidence.

**Supplementary Table I. State correspondence between metasurface unit cells**

**and diode ON/OFF configurations**

| States | PIN Diodes | |
| --- | --- | --- |
|  | PIN 3 | PIN 1 / PIN 2 |
| 00 | OFF | OFF |
| 01 |  | ON |
| 10 | ON | OFF |
| 11 |  | ON |

As depicted, Fig. S2 depicts the bottom-layer configuration of the proposed metasurface unit cell, functioning as a reflective phase shifter. Simultaneous switching of PIN1 and PIN2 introduces a 90° phase shift in reflection between their ON and OFF states. The sector-shaped stub connected to PIN3 acts as a short circuit at the operating frequency, enabling an additional 180° phase shift through PIN3 switching. Supplementary Table I summarizes the operational state mappings of the metasurface unit cell corresponding to different diode switching configurations.

As illustrated in Fig. S3, the reflection phases of the metasurface unit cell is simulated under normal incidence in CST Microwave Studio 2023. It can be observed that the reflection amplitude is larger than －2 dB, and phase differences between adjacent states of the 2-bit metasurface unit cell are approximately 90° across various reception angles at 5 GHz.

**Supplementary Note 5. The principle of decoupled angle and range estimation**

Regarding the coding scheme proposed in the text, according to (5) in the main body, the necessary and sufficient condition for the existence of two points$(\theta_{1},r_{1})$ and $(\theta_{2},r_{2})$ in the angle-range space with identical electric fields is:

$$\begin{aligned} 2\pi f_{c}\left( t-\frac{r_{1}}{c} \right)+\phi\left( t-\frac{r_{1}}{c}+\left( n-1 \right)\Delta t \right)+k_{0}d\left( n-1 \right)\sin\theta_{1} \\ =2\pi f_{c}\left( t-\frac{r_{2}}{c} \right)+\phi\left( t-\frac{r_{2}}{c}+\left( n-1 \right)\Delta t \right)+k_{0}d\left( n-1 \right)\sin\theta_{2}+2M\pi\\ n=1,2,3\ldots N,N\in Z\#\text{(S9)} \end{aligned}$$

This can be simplified to:

$$\begin{aligned} \frac{f_{c}}{c}\left( r_{2}-r_{1} \right)+\frac{[\phi\left( t-\frac{r_{1}}{c}+\left( n-1 \right)\Delta t \right)-\phi\left( t-\frac{r_{2}}{c}+\left( n-1 \right)\Delta t \right)]}{2\pi}=M-\frac{k_{0}d\left( n-1 \right)}{2\pi}(sin\theta_{1}-\sin\theta_{2}) \\ n=1,2,3\ldots N,N\in Z\#\text{(S10)} \end{aligned}$$

Since the right-hand side of the equation is independent of time *t*, for the equation to hold, the left-hand side must also be independent of *t*. However, according to the requirements for energy scanning, $\phi(t)$ is a time-varying signal. **Consequently, no two distinct points** $(\theta_{1},r_{1})$ **and** $(\theta_{2},r_{2})$ **can be found that satisfy condition (S10).**

**(a)	(b)**

Fig. S4. **Correlation analysis of received signals at different positions.** (a) Correlation coefficient as a function of angular separation Δθ for two points at the same range (r = 1 m). The reference point is fixed at (0°, 1 m). (b) Correlation coefficient as a function of range separation Δr for two points at the same angle (θ = 0°). The reference point is fixed at (0°, 1 m).

Fig. S4 shows the cross-correlation coefficient between the received electric fields at different positions  $(\theta_{i},r_{i})$ and  $(\theta_{j},r_{j})$ based on coding sequence in Fig. 4c. The reference point is taken as  $\left( \theta_{ref},r_{ref} \right)=(0^{\circ},1 m)$.

Figure S4(a) presents the correlation coefficient as a function of the angular separation $\Delta\theta$ for two points with the same range. It can be observed that when $\Delta\theta\neq0$, the correlation coefficient is always less than 1. When $\left| \Delta\theta\right|>10^{\circ}$, the correlation coefficient drops below 0.85. It is worth noting that MUSIC, as a super-resolution algorithm, can theoretically resolve two sources as long as their steering vectors are not completely coherent (i.e., the correlation coefficient is less than 1). To ensure experimental reliability, we constrained the angular positioning range between $-20^{\circ}$and $20^{\circ}$, and selected $10^{\circ}$ as the angular separation between adjacent target points.

Figure S4 (b) shows the correlation coefficient as a function of the range separation $\Delta r$ for two points with a fixed angular separation. The results indicate that the correlation decreases as the range separation increases. Considering the limitations of transmit power and the experimental site, we chose 5 m as the range separation between the target points.

**Supplementary Note 6. Reference-Based Calibration for Range Estimation**

Range estimation exhibits more noticeable errors. We have attempted to quantify the worst-case impact of this transition-induced phase error on ranging accuracy. A 3 ns timing error translates to a range error of approximately Δr=c×Δt=0.9 m. To mitigate this, we adopt a comparison method. Before any target measurement, a library of reference waveforms is pre-acquired using a calibration target placed at a known distance of 1 m. Since the waveform shape also depends on the target angle, reference waveforms are collected across a grid of angles from -20° to 20° in 10° steps.

When measuring an unknown target, the received waveform is first processed by the MUSIC algorithm to estimate the target angle. Based on this estimated angle, the corresponding reference waveform is retrieved from the pre-acquired library. The received waveform is then cross-correlated with this angle-matched reference waveform. The fixed time offset introduced by diode switching is present in both the measurement and the reference, and is therefore canceled out during cross-correlation. The peak position of the correlation result gives the relative time delay between the target and the reference distance, from which the target distance is calculated as R=1m+c⋅τpeak.

The reference-based calibration is primarily beneficial for short-range measurements, where the 0.9 m worst-case error would otherwise dominate. For mid-to-long-range sensing applications, however, the relative error becomes small even without calibration — to 3% at 10 m and 0.6% at 50 m — and the system remains highly reliable in those scenarios without the comparison method.

**Supplementary Note 7.** **Signal-to-Noise Ratio Analysis**

The SNR is calculated by comparing the average power of the received signal with the noise floor. The signal power is obtained by subtracting the noise power (measured with the metasurface moved away) from the total power (measured with the metasurface active). The measured SNR values at representative target positions are as follows:

Supplementary Table II. Signal-to-Noise Ratio at different positions

| Target Position | SNR |
| --- | --- |
| (0°, 1 m) | 43.54 dB |
| (0°, 20 m) | 22.70 dB |
| (20°, 1 m) | 38.12 dB |

Under all tested conditions, the SNR is sufficiently high for the MUSIC algorithm to produce clear spectral peaks and achieve stable joint range-angle estimation.
